# Radiofrequency ablation of papillary thyroid microcarcinoma

**DOI:** 10.3389/fsurg.2026.1828150

**Published:** 2026-06-10

**Authors:** Alessandro Serblin, Roberto Valcavi

**Affiliations:** The Endocrine and Thyroid Clinic, Reggio Emilia, Italy

**Keywords:** active surveillance (AS), flexible fiberoptic laryngoscopy (FFL), papillary thyroid microcarcinoma (PTMC), radiofrequency ablation (RFA), surgery, thyroid

## Abstract

Papillary thyroid microcarcinoma (PTMC), defined as papillary thyroid carcinoma measuring ≤1 cm, has emerged as a distinct clinical entity with a generally indolent course. The optimal management of PTMC remains controversial, with three main strategies currently adopted: surgical resection, active surveillance, and image-guided thermal ablation ( 16). This review provides a comprehensive analysis of these approaches, emphasizing their indications, outcomes, and limitations. Conventional surgery remains the gold standard, ensuring complete tumor excision but often associated with overtreatment, postoperative complications, and quality-of-life impairment. Active surveillance has gained acceptance as a conservative option for low-risk PTMC, reducing unnecessary interventions yet requiring long-term follow-up and patient compliance. Among minimally invasive alternatives, radiofrequency ablation (RFA) has shown growing evidence as a safe and effective technique for selected patients, achieving local tumor control with minimal morbidity and favorable cosmetic results. The review critically compares the efficacy, safety profiles, and patient selection criteria across these management modalities, highlighting the role of RFA as an emerging therapeutic tool that bridges the gap between surgical and observational strategies in the individualized treatment of PTMC.

## 1 Introduction

Papillary thyroid microcarcinoma (PTMC), defined as papillary thyroid carcinoma measuring 10 mm or less, has become one of the most frequently diagnosed thyroid malignancies worldwide. The incidence of PTMC has increased significantly over the past three decades, largely attributed to the widespread availability of high-resolution neck imaging techniques, particularly ultrasonography, and the routine use of ultrasound-guided fine-needle aspiration biopsy (US-FNAB). Recent data indicate that PTMC accounts for approximately 30.5% of all resected malignant thyroid tumors. The incidence of incidental PTMC (discovered during thyroidectomies for benign disease) has doubled from 4.5% to 9.0% over a seven-year period, while non-incidental PTMC (diagnosed preoperatively) has shown a tenfold increase, from 0.9% to 10.8%. Notably, this marked rise in incidence has not been accompanied by a proportional increase in mortality, which remains low at approximately 0.5 per 100,000 individuals, highlighting the indolent nature of this malignancy. Globally, thyroid cancer incidence is projected to increase by 29.9% by 2040, with a more pronounced rise in mortality expected primarily in low Human Development Index countries (1).

**Figure 1:**
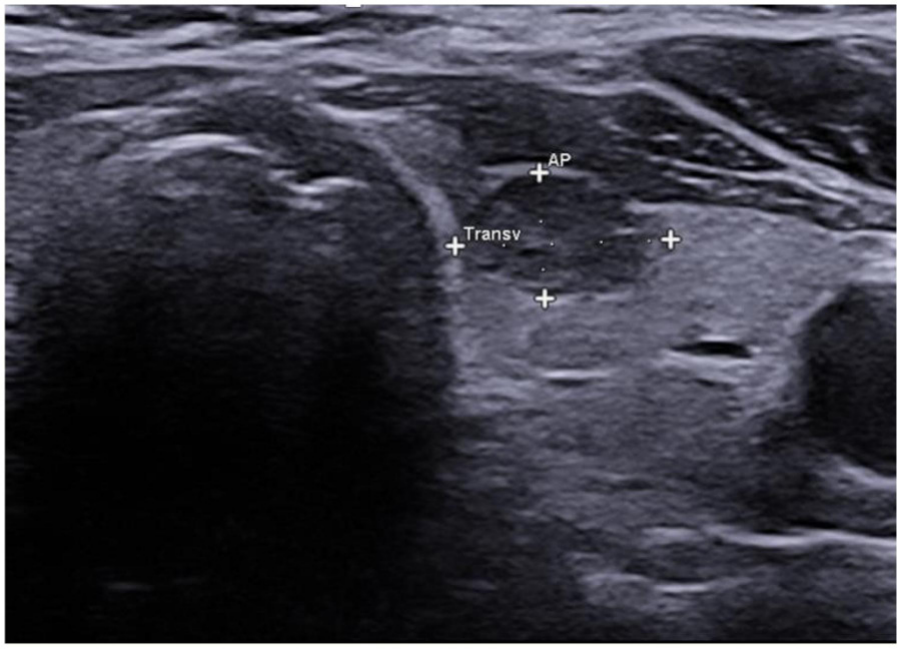
Unifocal PTMC of the left lobe.

The diagnosis of PTMC relies primarily on neck ultrasonography (Figure 1) and US-guided fine-needle aspiration biopsy (US-FNAB), which is considered the gold standard for cytological assessment and classification of malignancy risk. According to the American Thyroid Association (ATA) guidelines, nodules ≥10 mm with high to intermediate suspicious ultrasound patterns warrant FNA, though the clinical relevance of smaller nodules has led to refinements in diagnostic algorithms (2).

Sonographic features suggestive of malignancy include solid composition, hypoechogenicity, microcalcifications, irregular margins, and vertical growth pattern (taller-than-wide appearance). US-FNAB demonstrates high diagnostic sensitivity regardless of nodule size, although satisfactory specimen rates may be lower for nodules ≤5 mm. Core needle biopsy (CNB) has emerged as a complementary diagnostic tool, particularly for cases with inconclusive cytology or for post-treatment surveillance (3, 4).

Current management strategies for PTMC include three main approaches: surgical resection, active surveillance (AS), and minimally invasive thermal ablation techniques (5).

Lobo-isthmectomy represents the recommended surgical approach for unifocal PTMC without extrathyroidal extension or nodal metastases. Surgery provides complete tumor excision and histopathological confirmation, including evaluation of tumor multifocality, extrathyroidal extension, aggressive histological features, and lymph node involvement. However, thyroidectomy may be associated with overtreatment of clinically insignificant tumors, perioperative complications (including recurrent laryngeal nerve injury and hypoparathyroidism), potential loss of thyroid function requiring lifelong hormone replacement, and cosmetic concerns related to surgical scarring (3).

Active surveillance has gained increasing acceptance as a conservative management strategy for low-risk PTMC, based on seminal long-term studies demonstrating its safety in patients without aggressive features. This approach involves regular clinical and ultrasound monitoring without immediate intervention. Studies have shown that linear tumor growth may occur in approximately 8% of patients under AS, and a minority—particularly younger individuals—may develop cervical lymph node metastases over time, potentially requiring more extensive surgery. Notably, 8%–32% of patients under AS eventually undergo surgery, often due to anxiety associated with harboring an untreated malignancy rather than tumor progression (3, 6, 7).

Ultrasound-guided minimally invasive treatments (MIT), including radiofrequency ablation (RFA), laser thermal ablation (LTA), and microwave ablation (MWA), have emerged as promising non-surgical alternatives for low-risk PTMC. These procedures selectively ablate tumors without requiring general anesthesia, hospitalization, or resulting in thyroid function loss. Among thermal ablation modalities, RFA has demonstrated favorable oncologic outcomes in multiple studies, with pooled 5-year follow-up data revealing no tumor progression. Key advantages of MIT over surgery include lower procedural costs, reduced use of surgical resources, minimal work disruption, lower risk of major complications, preservation of thyroid function, and absence of visible scars. Compared to active surveillance, MIT offers definitive tumor eradication, potentially alleviating patient anxiety and reducing the risk of disease progression or delayed surgical intervention.

However, limitations of thermal ablation persist. Long-term prospective data confirming histologically complete tumor ablation remain scarce, and the technical challenges of achieving oncologically radical treatment increase with tumor size and unfavorable location (proximity to trachea, major vessels, or recurrent laryngeal nerve). Furthermore, post-ablation sonographic changes may hinder detection of residual or recurrent disease, necessitating continued long-term surveillance (6–8).

The choice among surgery, active surveillance, and thermal ablation requires a multidisciplinary approach, integrating clinical and ultrasound staging, patient preferences, comorbidities, and local expertise. The European Thyroid Association and international consensus statements recommend that MIT be considered in experienced centers for patients who are not surgical candidates or who refuse surgery while seeking treatment to minimize the risk of disease progression. Comprehensive patient counseling on the benefits and limitations of each modality is essential to ensure informed shared decision-making (6).

Ultrasound-guided percutaneous ablation may be considered as an alternative to active surveillance or resection for cT1aN0M0 PTC in selected patients. Shared clinical decision-making between the patient and clinical team regarding risks and benefits of this approach is essential.[]

## 2 Thermal ablation technologies for PTMC

Thermal ablation techniques represent a paradigm shift in the management of papillary thyroid microcarcinoma (PTMC), offering minimally invasive alternatives to surgery by inducing localized hyperthermia to achieve tumor necrosis. These ultrasound-guided procedures, as you can see in Table 1, including laser ablation (LA), high-intensity focused ultrasound (HIFU), microwave ablation (MWA), and radiofrequency ablation (RFA), have demonstrated comparable efficacy in volume reduction and low recurrence rates, with pooled complete disappearance rates around 57.6% and recurrence at 0.4% across modalities. Systematic reviews confirm their safety, with overall complication rates of 3.2% and major complications at 0.7% (9).

**Table 1:** Thermal ablation technologies for PTMC.

Ablation Technique	Mechanism Principle	Key Procedural Considerations	Primary Indications
Radiofrequency Ablation (RFA)	Uses high frequency alternating electric current to generate frictional heat (up to 100 °C), leading to coagulation necrosis.	Transisthmic approach utilizing a moving shot technique. Uses an internally cooled electrode needle. Efficacy may be limited by tissue carbonization and heat sink effectfrom adjacent blood flow or cystic components. Typically performed under local analgesia.	Symptomatic benign nonfunctioning nodules (pooled volume reduction 76.1% at 6 months; 80.3% at 3 years). May be considered for small autonomously functioning nodules (AFNs) (normalization rate up to 90.9%) and Papillary Thyroid Microcarcinoma (PTMC).
			Larger nodule has higher absolute volume reduction (15).
Laser Ablation (LA)	Delivers focused light energy through flexible optical fibers, converted into heat (up to 100 °C) via photon scatter, causing coagulation necrosis.	Transisthmic approach. Flexible optical fibers are inserted and then withdrawn incrementally while administering energy pulses. Delivers the lowest total energy among thermal ablative techniques, potentially conferring greater safety and control in critical areas. Typically performed under local analgesia.	Symptomatic benign nonfunctioning nodules (pooled volume reduction 48.3–49.9% at 6 months). More effective in spongiform and mixed nodules compared with solid nodules. May be considered for small AFNs and PTMC.
Microwave Ablation (MWA)	Uses an electromagnetic field causing oscillation of polarized particles to generate heat through particle collision. Results in high tissue temperature.	Transisthmic moving shot technique, treating ablation units with 5–10 s of microwave pulses. The generated heat is not impeded by char or heat sink. This characteristic may pose a potential greater risk of injury near critical structures in the central neck. Typically performed under local analgesia.	Symptomatic benign nonfunctioning nodules (volume reduction 88.6–92.4% at 12 months). May be considered for PTMC. Minimal data exists for AFNs and recurrent thyroid cancer.
High-Intensity Focused Ultrasound (HIFU)	Focuses high-intensity sound waves from numerous sources onto a target, causing tissue vibration and frictional heat (up to 85 °C).	Completely noninvasive technique without needle puncture. A cooled probe is positioned on the skin; HIFU pulses are delivered followed by cooling time. Typically requires general anesthesia, unlike other ablation techniques. Efficacy may be limited by motion. Longest procedure time (45–60 min).	Symptomatic benign nonfunctioning nodules (volume reduction 48.7–68.9% at 6–12 months). Minimal data exists for other indications. Associated with shorter length of stay and fewer complications compared to surgery.

Laser ablation employs an Nd:YAG or diode laser fiber inserted into the tumor under ultrasound guidance to deliver precise thermal energy, leading to coagulative necrosis. It is particularly suitable for small PTMC (<10 mm) in high-risk surgical patients, as evidenced by case reports showing complete tumor ablation without recurrence at 24-month follow-up. Meta-analyses indicate LA achieves a volume reduction rate (VRR) of approximately 88.6%, though it is less effective than other modalities in complete disappearance due to smaller treatment zone (9, 10).

HIFU is a completely non-invasive technique that uses extracorporeal ultrasound beams to focally heat the target lesion without needle puncture. Limited studies on PTMC report feasibility with significant volume reduction, but its deeper penetration and precision are offset by challenges in treating superficial or irregular nodules. Evidence remains preliminary compared to percutaneous methods, with fewer multicenter trials available (11, 12).

MWA generates rapid heating through microwave energy via a needle antenna, producing larger ablation zones than RFA in shorter times. For low-risk PTMC, MWA yields high complete absorption rates and VRR up to 95.3%, with histopathological findings of fibrous hyperplasia post-treatment. Network meta-analyses show MWA with the lowest recurrence probability among thermal ablations, though it may involve slightly higher energy delivery (9).

RFA uses a needle electrode to create an alternating current field, generating frictional heat up to 100 °C with hydrodissection or moving-shot techniques to protect adjacent structures. It demonstrates superior outcomes in PTMC, with 5-year VRR of 99.94%, complete disappearance in 92%–100% of cases, and negligible recurrence (0%–0.4%) at long-term follow-up (up to 60 months). RFA excels in reduced operation time, blood loss, hospital stays, and complications compared to surgery and other ablations, positioning it as the most studied and versatile option (13, 14).

This is an overview of the main thermal ablation techniques for thyroid nodules described above:

## 3 Indications and procedural technique for RFA

Radiofrequency ablation (RFA) is indicated for low-risk PTMC defined as solitary nodules ≤1 cm without extrathyroidal extension, lymph node metastasis, or aggressive histological features, particularly in patients unsuitable for surgery or preferring minimally invasive options (16). According to consensus guidelines from the Korean Society of Thyroid Radiology, ideal candidates include those with high surgical risk, refusal of surgery despite active surveillance concerns, bilateral multifocal PTMC, or recurrence post-surgery (17). Contraindications encompass pregnancy, uncorrectable coagulopathy, and tumors adjacent to critical structures like the recurrent laryngeal nerve without hydrodissection feasibility (9, 11).

**Figure 2:**
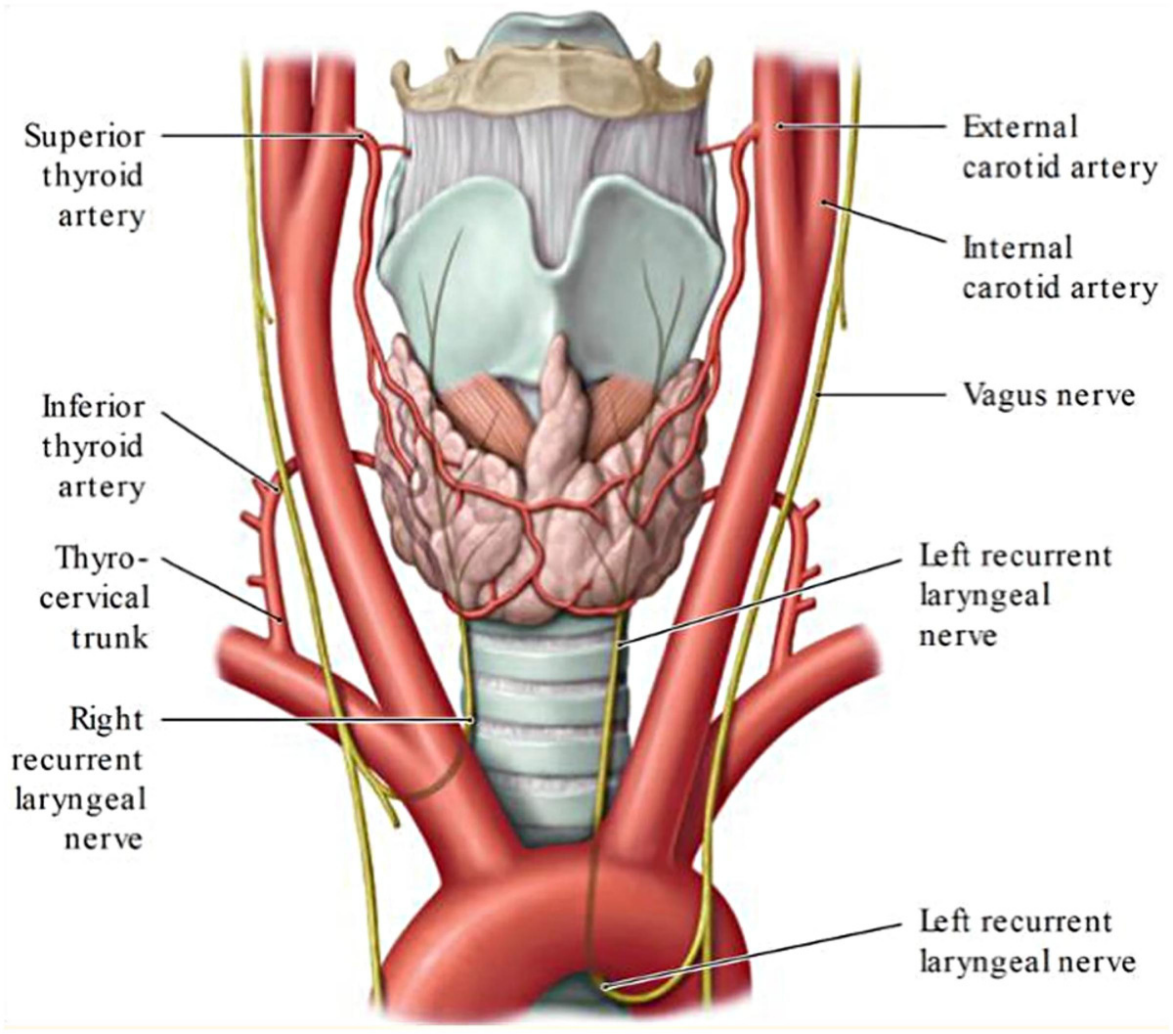
Recurrent laryngeal nerve anatomy.

RFA procedures are performed under local anesthesia with ultrasound guidance, employing monopolar or bipolar systems and standardized approaches such as the moving-shot technique (MST) or trans-isthmic approach for complete ablation. During thyroid radiofrequency ablation (RFA), it is crucial to protect the recurrent laryngeal nerves (RLNs) and the external branch of the superior laryngeal nerve (EBSLN) from thermal damage. These nerves are essential for vocal function, and the RLNs, in particular, are at high risk due to their proximity to the thyroid gland. The RLNs, which branch off the vagus nerve, follow a complex path. The left nerve loops under the aortic arch, while the right one circles the subclavian artery before both ascend in the groove between the trachea and esophagus, situated directly behind the thyroid gland (Figure 2) (18).

**Figure 3:**
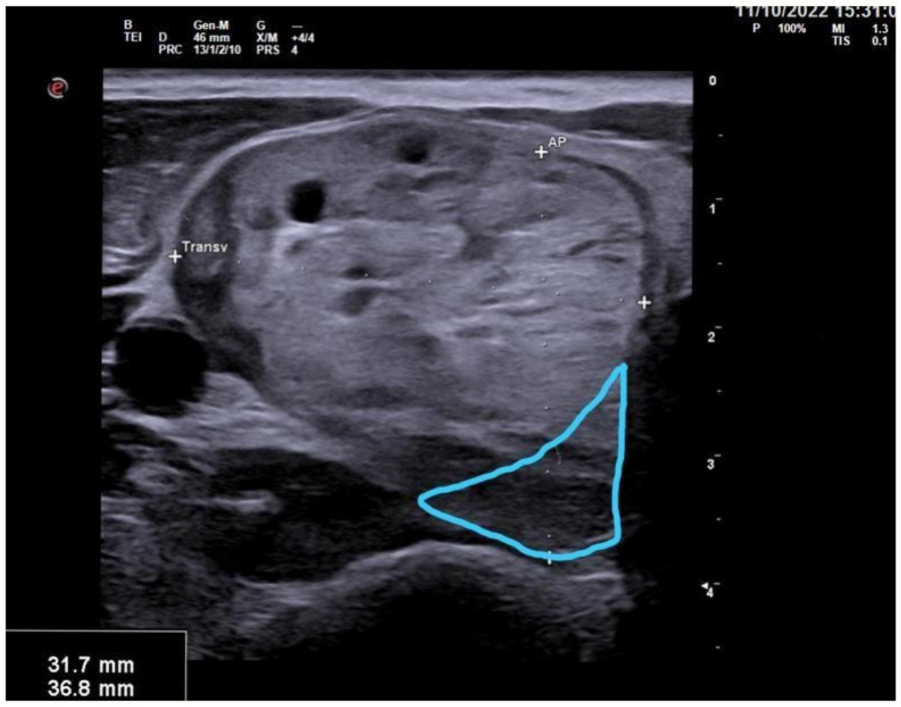
Thedangerzone/triangle.

This area is often referred to as the “danger zone” because the nerves cannot be seen on ultrasound (Figure 3).

It is also important to remember that these nerves have anatomical variations. In nearly 40% of people, the RLN splits into multiple branches before reaching the larynx. This makes the “danger zone” even more complex and highlights the need for extreme caution.

Damage to the RLNs and EBSLN can lead to hoarseness, difficulty speaking, and a change in voice pitch. More severe injury can weaken the laryngeal muscles, causing swallowing difficulties (dysphagia) and increasing the risk of aspiration. While some researchers believe that this nerve damage can be temporary, complete recovery of vocal quality and muscle strength is not guaranteed. Therefore, preventing injury is the primary goal.

The most effective way to protect these nerves is through a technique called hydrodissection. This involves injecting fluid between the thyroid nodule and the surrounding structures. This creates a protective buffer that separates the nerves from the heat generated by the RFA procedure, significantly lowering the risk of thermal injury. If nerve injury is suspected during RFA—for example, if the patient's voice suddenly changes the procedure should be stopped immediately and a “rescue” procedure should be performed. Due to its small diameter, RLN cannot be visualized by ultrasound. However, using US images, we can determine that RLN is positioned in the so-called “danger area”, posterior and medial to the thyroid lobe (19).

## 4 Rescue hydrodissection

**Figure 4:**
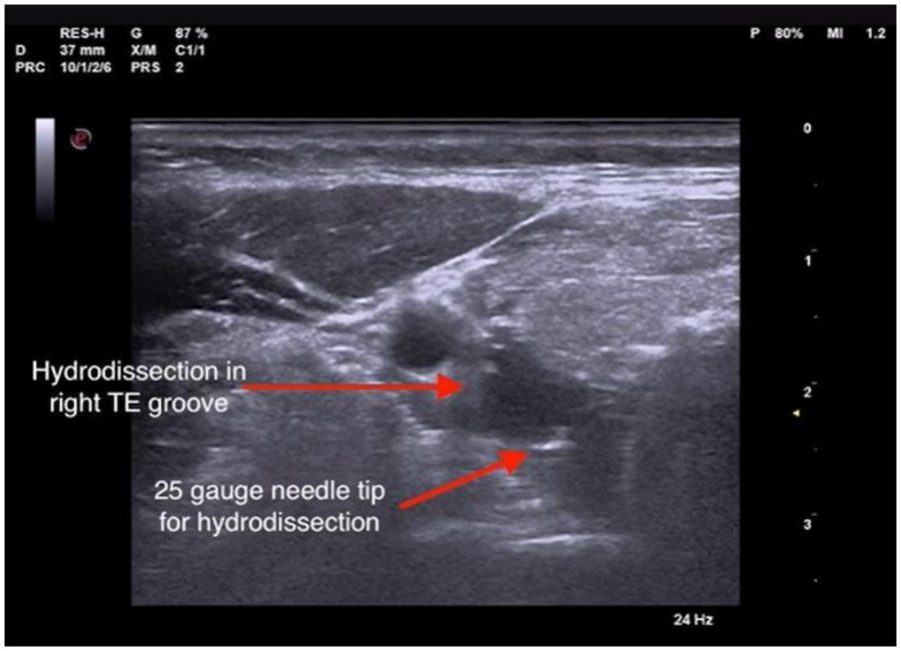
RLN rescue hydrodissection.

Knowledge of the recurrent laryngeal nerve (RLN), external branch of the superior laryngeal nerve (EBSLN), vagus nerve, cervical sympathetic ganglion, cervical and brachial plexuses, spinal accessory nerve, and phrenic nerve is clinically vital for RFA procedures. A strong understanding of the location and function of these nerves allows for the prompt detection, prevention, and correct management of potential complications. These complications can present in various ways, including hoarseness, alterations in voice projection, tone, and pitch, as well as tachycardia, irregular heart rhythms, low blood pressure, vomiting, Horner's syndrome, arm numbness, paralysis, a drooping shoulder, and hiccups (20, 21). Should thermal damage to a nerve structure occur, injecting cold D5W (5% dextrose in water) is a straightforward yet effective treatment that can lead to a quick resolution of symptoms. A specific “rescue” procedure has been developed for the RLN if a patient experiences hoarseness during thyroid RFA. If hoarseness is noticed, the procedure must be immediately halted, and cold D5W should be injected into the tracheoesophageal groove. This action typically lessens thermal injury to the RLN, and the patient's voice quality usually returns to normal quickly. Given the serious consequences of nerve damage, all RFA operators should be trained in this “rescue” procedure and have cold D5W readily available for all procedures (Figure 4) (22).

## 5 Vocal cord monitoring by flexible fiberoptic laryngoscopy (FFL) during thyroid RFA

**Figure 5:**
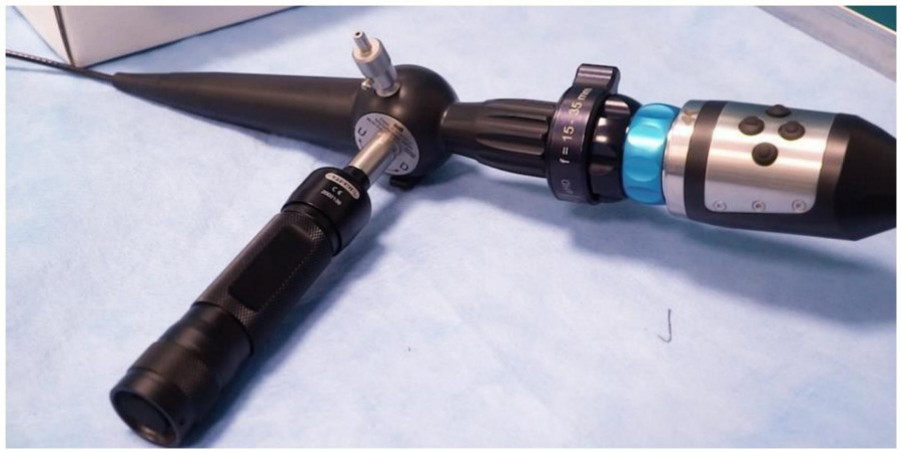
Flexible fiberoptic laryngoscopy (FFL).

Thermal injury to RLN during RFA may produce temporary or permanent vocal cords paralysis. A non-invasive diagnostic test compared to laryngoscopy for the assessment of the vocal cords is transcutaneous laryngeal ultrasound (TLUSG) (23); however, the gold standard for laryngeal motility assessment is Fiberoptic Fibrolaryngoscopy (FFL) (Figure 5) (19, 24).

**Figure 6:**
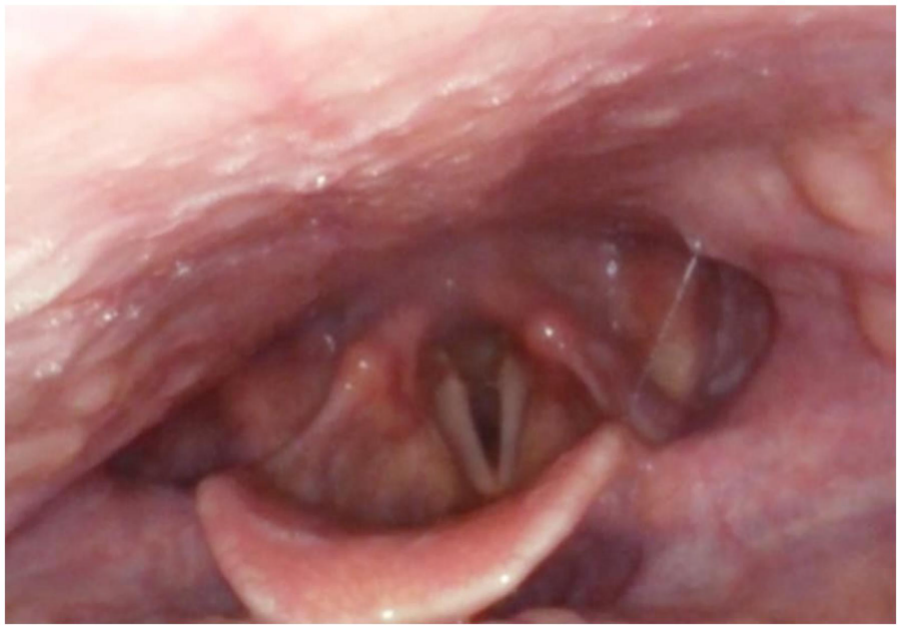
FFL with visualization of the larynx and vocal cords.

FFL allows RLN assessment by visualizing vocal cord symmetrical movements during calm breathing (Figure 6). FFL monitoring during RFA may detect RLN disfunction if vocal cord asymmetry is detected.

FFL is preferably used for ablations of large thyroid nodules or thyroid nodules near the danger triangle. It is also strongly recommended for specific professional groups such as voice professionals (singers, actors and TV broadcasters), where the protection of RLNs and EBSLNs is of utmost importance. FFL is easily performed by an ENT specialist and well tolerated by the patient.

FFL and vocal sounds produced by patient allow RLN and EBSLN assessment by visualizing of vocal cord symmetrical movements. Should vocal cord asymmetry appear during RFA, the procedure is immediately stopped and hydrodissection with cold D5W is performed. Through FFL monitoring, vocal cord symmetrical movements during calm breathing can be observed. A series of postoperative FFLs are performed on patients after RFAs to confirm normal vocal cord symmetric adduction during phonation and abduction during breathing (19, 25).

## 6 Clinical outcomes and complications

RFA achieves excellent oncologic control in PTMC, with systematic reviews reporting volume reduction rates (VRR) exceeding 90% at 12 months and complete disappearance in 49%–100% of cases at 5 years. A retrospective study of 445 PTMC patients showed no regrowth or new lesions at mean 39.3-month follow-up, with lymph node metastasis in only 0.7%. Network meta-analyses rank RFA highly for complete response, surpassing laser and HIFU while comparable to MWA. Long-term data (up to 7 years) confirm sustained efficacy without increased mortality (13, 26, 27).

Major complication rates for RFA in PTMC are low at 0%–1.3%, primarily transient voice changes (0.4%–1.1%) due to perithyroidal nerve irritation, managed conservatively. Minor issues include pain, hematoma, and skin burns (1%–3%), with no permanent hypoparathyroidism or hypothyroidism. Compared to surgery, RFA reduces overall morbidity while preserving quality of life (9, 10).

Prospective multicenter randomized controlled trials are essential to establish long-term oncologic outcomes of RFA beyond 5–7 years and to compare it directly with surgery and active surveillance in diverse populations. Technological advancements, such as enhanced ultrasound imaging, real-time temperature monitoring, and artificial intelligence for patient selection, promise to improve precision and standardization. Standardized response-to-ablation criteria (complete, indeterminate, incomplete) will refine post-procedural surveillance and predict local tumor progression risk (27–29).

Thermal ablation, including RFA, demonstrates superior economic benefits over surgery for PTMC, with significantly lower costs (SMD-3.40), shorter hospitalization (SMD-3.82 days), and reduced operation times (SMD-5.67 min), without compromising efficacy or safety. Compared to active surveillance (AS), RFA is cost-effective at $23,821/QALY, particularly for anxious patients opting out of observation. AS remains more cost-effective for elderly patients (>60 years), but RFA offers value in younger cohorts or those preferring intervention (30–32).

RFA is recommended by international consensus for unifocal, low-risk PTMC (<1 cm) in non-surgical candidates or those refusing surgery, performed in high-volume centers by experienced operators. It serves as step-up therapy post-AS tumor growth or initial treatment for bilateral/multifocal disease. Multidisciplinary evaluation, shared decision-making, and lifelong ultrasound follow-up are mandatory (33–35).

## 7 Conclusion

Radiofrequency ablation (RFA) has established itself as a safe, effective, and minimally invasive treatment for low-risk papillary thyroid microcarcinoma (PTMC), achieving high rates of complete tumor disappearance (up to 100%) and negligible recurrence (0%–0.4%) with low complication profiles. Compared to traditional surgery, RFA offers superior cosmesis, thyroid function preservation, and reduced morbidity, while surpassing active surveillance in providing definitive tumor control and alleviating patient anxiety (11, 13, 27).

Cost-effectiveness analyses further support its integration into clinical practice, particularly for non-surgical candidates, with substantial savings over lobectomy. However, RFA should be reserved for experienced centers, as long-term prospective data and standardized protocols are needed to solidify its role against emerging thermal ablation competitors like MWA (9, 30).

In summary, RFA bridges the gap between overtreatment and undertreatment in PTMC management, promoting personalized care through multidisciplinary decision-making. Future randomized trials will refine indications and optimize outcomes, potentially reshaping thyroid oncology paradigms (27, 33, 34).
